# Comparative genomics of *Enterococcus faecalis *from healthy Norwegian infants

**DOI:** 10.1186/1471-2164-10-194

**Published:** 2009-04-24

**Authors:** Margrete Solheim, Ågot Aakra, Lars G Snipen, Dag A Brede, Ingolf F Nes

**Affiliations:** 1 Laboratory of Microbial Gene Technology and Food Microbiology, Department of Chemistry, Biotechnology and Food Science, The Norwegian University of Life Sciences, N-1432 Ås, Norway; 2 Section for Biostatistics, Department of Chemistry, Biotechnology and Food Science, The Norwegian University of Life Sciences, N-1432 Ås, Norway

## Abstract

**Background:**

*Enterococcus faecalis*, traditionally considered a harmless commensal of the intestinal tract, is now ranked among the leading causes of nosocomial infections. In an attempt to gain insight into the genetic make-up of commensal *E. faecalis*, we have studied genomic variation in a collection of community-derived *E. faecalis *isolated from the feces of Norwegian infants.

**Results:**

The *E. faecalis *isolates were first sequence typed by multilocus sequence typing (MLST) and characterized with respect to antibiotic resistance and properties associated with virulence. A subset of the isolates was compared to the vancomycin resistant strain *E. faecalis *V583 (V583) by whole genome microarray comparison (comparative genomic hybridization (CGH)). Several of the putative enterococcal virulence factors were found to be highly prevalent among the commensal baby isolates. The genomic variation as observed by CGH was less between isolates displaying the same MLST sequence type than between isolates belonging to different evolutionary lineages.

**Conclusion:**

The variations in gene content observed among the investigated commensal *E. faecalis *is comparable to the genetic variation previously reported among strains of various origins thought to be representative of the major *E. faecalis *lineages. Previous MLST analysis of *E. faecalis *have identified so-called high-risk enterococcal clonal complexes (HiRECC), defined as genetically distinct subpopulations, epidemiologically associated with enterococcal infections. The observed correlation between CGH and MLST presented here, may offer a method for the identification of lineage-specific genes, and may therefore add clues on how to distinguish pathogenic from commensal *E. faecalis*. In this work, information on the core genome of *E. faecalis *is also substantially extended.

## Background

Enterococci are Gram-positive facultative anaerobic cocci with a low GC-content. They are natural inhabitants of the mammalian gastrointestinal (GI) tract and among the first lactic acid bacteria to colonize the intestines of a newborn [1]. During the last three decades, enterococci have emerged as important pathogens and as a major cause of nosocomial infections. The majority of hospital-acquired, enterococcal infections is caused by *Enterococcus faecalis *[2]. Several putative virulence factors have been characterized in *E. faecalis *(reviewed in [2]), and their roles in pathogenicity have been established in various animal models [3-6] and cultured cell lines [7,8]. A large number of reports on enterococcal pathogenicity has focused on the presence or absence of these virulence determinants in enterococcal isolates from different origins [9-14]. The results have shown that several of the putative virulence traits are detected in enterococcal isolates independent of their origin, suggesting that these factors may not be crucial for enterococcal pathogenicity. However, a higher incidence of some of the virulence determinants in clinical isolates may indicate that these genes enhance the ability of *E. faecalis *to cause disease, as suggested by virulence studies on bacterial mutants in animal models [3].

The sequencing of the *E. faecalis *V583 genome (V583) [15] made global analyses of whole genome diversity within this species possible [16-18], by the use of microarray-based comparative genomic hybridization (CGH). The approximate size and composition of the *E. faecalis *core genome have been investigated on clinical, food and environmental isolates [17,18]. The CGH-approach has also been used to evaluate the dissemination of variable traits from the V583 genome within diverse lineages of the species [17]. Previous analyses have shown that the main genomic variation between the strains correspond to previously identified mobile genetic elements (MGEs) in V583 [17,18]. However, an effort to explore the gene content of commensal *E. faecalis *by CGH has not previously been made and little is known about genetic determinants that may explain the differences in life style between pathogenic and non-pathogenic *E. faecalis *strains.

The aim of this study was to investigate the genomic diversity among fecal *E. faecalis *isolated from healthy Norwegian infants by means of CGH. In an attempt to study genetic variability of commensal *E. faecalis *isolates, we used genome-wide DNA arrays to probe the presence of 3219 open reading frames (ORFs) from V583 and 10 ORFs representing a 17-kb deletion in the V583 pathogenicity island in our collection of community-derived Norwegian fecal *E. faecalis *baby isolates. The isolates were also characterized with respect to antibiotic resistance and properties associated with virulence, by PCR and phenotypic assays.

## Methods

### Stool samples

This study included 11 healthy Norwegian infants (7 male and 4 female) born in the Eastern part of Norway. The babies were all born in Oslo and Akershus Counties. Informed consent had been obtained from their parents. From infants A-C, stool samples were collected once each month during the first six months and once after 12 months from October 2004 to September 2005, while from infants D-K, samples were collected once during the first six months of life during 2002. All the samples were collected after mother and child had left the obstetric ward. All the infants were born by vaginal delivery and all were breast-fed during the period of sampling. None of the infants were treated with antibiotics during the sampling period. A total of 29 stool samples were collected.

### Identification of enterococcal isolates and growth conditions

From each stool sample, two cultures were prepared: 1 g fecal material was homogenized in 1) 10 mL deMan-Rogosa-Sharpe (MRS; Oxoid) broth and 2) 10 mL Arroyo, Martin and Cotton broth (AMC; [19]), by vortexing. Serial dilutions were made and 100 μl of the 10^-5 ^– 10^-7 ^dilutions were plated on MRS- and AMC agar plates, respectively. MRS plates were incubated aerobically over night (ON) at 37°C, while AMC plates were incubated anaerobically ON at 37°C. Plates were then examined for growth, and colonies with different morphology and from different plates were picked and inoculated in 5 ml MRS- or AMC-broth, depending on which plates they were isolated from. The cultures were incubated as described above. Genomic DNA from each sample was isolated, and for identification, the 16S rRNA gene from each isolate was amplified and sequenced using general 16S rDNA primers (Additional file 1). PCR was accomplished using DyNAzyme™ II DNA Polymerase (Finnzymes). Thermocycling conditions were as follows: 2 min at 94°C; followed by 30 cycles of 30 s at 94°C, 30 s at 56°C, and 1.5 min at 72°C; followed by 10 min at 72°C. A total of 31 different *E. faecalis*-isolates were identified, and further analyzed in this study (Table 1). The 31 enterococcal isolates were isolated as part of two surveys of the content of lactic acid bacteria (LAB) in baby feces, hence, the culture media used were rich media, not particularly chosen to select for enterococci. In the present study, *E. faecalis *were grown aerobically ON in brain heart infusion broth (BHI; Oxoid) at 37°C.

**Table 1:** Fecal *Enterococcus faecalis *isolates used in this study.

**Infant**	**Isolate**	**MLST**		**CPS**	**Ab^R^**	**Genotypes**	**Phenotypes**
		**ST**	**CC**				
A	39A	91	S	-	-	*ace, agg, esp, fsrB, gelE*	GEL
A	88A	91	S	-	-	*ace, agg, esp, fsrB, gelE*	GEL
**A**	**92A**	**44**	**44**	**T1**	**-**	ace, agg *	**-**
**A**	**111A**	**161**	**8**	**T1**	**aT**	***ace, agg, cylL, esp, gelE***	**CYL**
A	112A	64	8	-	T	*ace, agg, cylL, esp, gelE*	CYL
A	123A	64	8	-	T	*ace, agg, esp, gelE*	CYL
A	125A	64	8	-	aT	*ace, agg, cylL, esp, gelE*	CYL
A	157A	91	S	-	-	*ace, agg, esp, fsrB, gelE*	GEL
B	2B	30	30	-	aT	*ace, agg, esp, gelE*	-
B	75B	30	30	-	a	*ace, agg, esp, gelE*	-
B	132B	44	44	-	T	*ace, agg, cylL, fsrB, gelE*	GEL
**B**	**158B**	**6**	**2**	**T2**	**a**	***ace, agg, cylL, fsrB, gelE***	**CYL**
B	226B	6	2	-	-	*ace, agg, cylL, fsrB, gelE*	CYL
C	26C	44	44	-	T	*ace, agg, cylL, fsrB, gelE*	GEL
**C**	**29C**	**44**	**44**	**T1**	**aT**	***ace, agg, cylL, fsrB, gelE***	**CYL GEL**
C	34C	44	44	-	T	*ace, agg, cylL, fsrB, gelE*	GEL
C	105C	194	S	-	T	*ace, agg, esp, fsrB, gelE*	GEL
C	141C	44	44	-	aT	*ace, agg, esp, fsrB, gelE*	GEL
D	4	30	30	-	a	*ace, esp, gelE*	-
E	59	30	30	-	a	*ace, esp, gelE*	-
**F**	**62**	**66**	**S**	**T1**	**T**	***ace, agg, esp, gelE***	**-**
**G**	**85**	**30**	**30**	**T5**	**AG**	***ace, esp, gelE***	**-**
**H**	**105**	**16**	**S**	**T2**	**aEGT**	***ace, agg, cylL, esp, fsrB, gelE***	**CYL GEL**
I	135	16	S	-	aEGT	*ace, agg, cylL, esp, fsrB, gelE*	GEL
I	137	30	30	-	a	*ace, esp, gelE*	-
I	236	16	S	-	EGT	*ace, agg, esp, fsrB, gelE*	GEL
J	173	55	55	-	aET	*ace, agg, cylL, esp*	-
**J**	**189**	**162**	**72**	**T5**	**a**	***ace, agg, cylL, fsrB, gelE***	**CYL GEL**
J	199	162	72	-	-	*ace, agg, cylL, esp, fsrB, gelE*	GEL
**K**	**266**	**163**	**S**	**T2**	**aT**	***ace, agg, fsrB, gelE***	**-**
K	267	163	S	-	-	*ace, agg, cylL, esp, fsrB, gelE*	GEL

* Isolate 92A was not genotyped for the presence of *gelE*, and *fsrB *by PCR.

The isolates from infants A-C are listed chronologically, according to their time of isolation. Isolates that have been genomotyped by CGH are listed in bold. CS; community surveillance, MLST; multilocus sequence typing, ST; sequence type, CC; clonal complex, S; singleton, CPS; capsular locus polymorphism type, Ab^R^; antibiotic resistance, A; ampicillin, E; erythromycin, G; gentamicin, T; tetracycline, *ace*; collagen-binding adhesin, *agg*; aggregation substance, *esp*; enterococcal surface protein, *frsB*;, *gelE*; gelatinase, *cylL*; cytolysin L, CYL; cytolysin activity, GEL; gelatinase activity.

### MLST analysis

MLST was performed according to the scheme presented by Ruiz-Garbajosa et al. [20], using the ABI Prism Big dye Cycle Sequencing Ready Reaction kit (Applied Biosystems) in an ABI PrismTM 3100 Genetic Analyzer. Sequence types were defined by the allelic variation at the seven loci *aroE*, *gdh*, *gki*, *gyd*, *pstS*, *xpt *and *yqiL*. Isolates with the same sequence type are thought to be members of a single clone or lineage. Clonal complexes were defined as groups of isolates that differed in no more than two of the seven loci analyzed, by use of eBURST [21]. Each clonal complex was designated after its ancestor sequence type (ST) or the representative ST that appeared with the highest frequency. All the MLST data from this study has been deposited into the *E. faecalis *MLST database .

### Phenotypic assays

#### Cytolysin assay

Hemolytic activity was qualitatively detected by the use of blood agar plates supplemented with 5% (v/v) defibrinated horse blood, 1% (w/v) glucose and 0.03% (w/v) L-arginine (Sigma) [22]. Overnight cultures were diluted 1:100, spotted onto fresh plates and incubated at 37°C for 24 h. Zones of clearing around colonies indicated production of cytolysin.

#### Gelatinase assay

Detection of gelatinase activity was performed by the use of Todd-Hewitt (Oxoid) agar plates containing 3% gelatin [23]. Overnight cultures were diluted 1:100, spotted onto fresh plates and incubated at 37°C overnight, before they were placed at 4°C for 5 h. Zones of turbidity around colonies indicated hydrolysis of gelatin.

#### Antimicrobial susceptibility testing

BHI agar plates supplemented with 4 μg/ml ampicillin, 20 μg/ml chloramphenicol, 50 μg/ml erythromycin, 500 μg/ml gentamicin, 10 μg/ml tetracycline or 4 μg/ml vancomycin were used. The plates were inoculated by spotting 5 μl (10^6^–10^7 ^CFU) overnight culture (1: 200) and incubated at 37°C overnight. Growth was interpreted as resistance to the antibiotic added to the medium.

#### Detection of genes encoding virulence factors and bacteriocin genes

The presence of *ace*, *agg*, *esp*, *cylL *and *gelE *were detected by means of polymerase chain reactions (PCR) as previously described [9,24]. Isolates were also tested for the presence of *iolE*, *iolR *and genes coding for the following bacteriocins by PCR: enterocin A (EA), enterocin B (EB), enterocin P (EP), enterolysin A (EN), enterocin L50 (EL50) and enterocin 1071A and B (E1071A&B). Thermocycling conditions were as follows: 2 min at 94°C; followed by 30 cycles of 30 s at 94°C, 30 s at 50 ± 10°C, and 30 s at 72°C; followed by 10 min at 72°C. Primers are listed in Additional file 1.

### API 50 CH for determination of fermentation patterns

Carbohydrate fermentation patterns were obtained for selected isolates with API 50 CH kits (BioMerieux) according to the manufacturer's instructions.

### Comparative genomic hybridization

#### Microarrays

The microarrays used in this work contained 3219 open reading frames from the genome of *Enterococcus faecalis *V583 [15] represented by oligonucleotides (70-mers; probes). Of these 3219 ORFs, 3093 were chromosomal ORFs and 126 were located on plasmids. In addition, ten genes from the pathogenicity island (PAI) of *E. faecalis *MMH594 (deleted in the PAI of V583) were represented [25]. The 70-mer oligos were supplied by Invitrogen. The oligos were spotted in triplicates onto epoxy-coated glass slides (Corning). In order to reduce biases due to positional effects, the replicate spots were spotted at random positions within a subarray on the array. Alien reporter sequences (SpotReport^®^Alien^® ^Oligo Array Validation System, Stratagene), without homology to any known nucleic acid sequences in public databases, were spotted as negative controls on the array. The microarray design has been deposited in the ArrayExpress database with the accession number A-MEXP-1069.

#### DNA isolation

For CGH, 9 isolates were chosen based on their representation of MLST sequence type diversity across the babies and of novel sequence types. Genomic DNA was isolated by using the FP120 FastPrep bead-beater (BIO101/Savent) and the QiaPrep MiniPrep kit (Qiagen) as follows: 10 mL overnight cultures were centrifuged for 5 min. at 6000 rpm in an Eppendorf 5804R tabletop centrifuge at 4°C, and pellets were resuspended in 250 μl RNaseA-containing Buffer P1 (100 μg/mL RNaseA). The cell suspensions were transferred to 2 mL screw cap FastPrep tubes (Qbiogene) containing 0.5 g acid-washed glass beads (< 106 μm) (Sigma). Cells were lysed by shaking the tubes for 20 s at 6 m/s in the FastPrep bead-beater. After a short spin, lysed cells were transferred to Eppendorf tubes and 250 μl Buffer P2 and 350 μl Buffer N3 was added to each tube. Then, the suspensions were centrifuged for 10 min. at 13000 rpm in a tabletop centrifuge (Biofuge Pico, Heraeus) at room temperature, before the supernatant fluids were loaded on to Qiaprep spin columns. The columns were washed again and genomic DNA was eluted according to the Qiaprep Spin MiniPrep kit protocol. Concentration and purity of the genomic DNA was measured using the NanoDrop spectrophotometer (NanoDrop Technologies). 5 μg genomic DNA was used for each labeling reaction.

#### Fluorescent labeling and hybridization

Genomic DNA was labeled and purified with the BioPrime Array CGH Genomic labeling System (Invitrogen) and Cyanine Smart Pack dUTP (PerkinElmer Life Sciences), according to the manufacturer's protocol.

Purified samples were then dried, prior to resuspension in 140 μl hybridization solution (5 × SSC, 0.1% (w/v) SDS, 1.0% (w/v) bovine serum albumin, 50% (v/v) formamide and 0.01% (w/v) single-stranded salmon sperm DNA) and hybridized for 16 h at 42°C to the *E. faecalis *oligonucleotide array in a Tecan HS 400 pro hybridization station (Tecan). Arrays were washed twice at 42°C with 2 × SSC + 0.2% SDS, and twice at 23°C with 2 × SSC, followed by washes at 23°C with 1) 0.2 × SSC and 2) H_2_O. Two replicate hybridizations (dye-swap) were performed for each test strain. Hybridized arrays were scanned at wavelengths of 532 nm (Cy3) and 635 nm (Cy5) with a Tecan scanner LS (Tecan). Fluorescent intensities and spot morphologies were analyzed using GenePix Pro 6.0 (Molecular Devices), and spots were excluded based on slide or morphology abnormalities. All water used for the various steps of the hybridization and for preparation of solutions was filtered (0.2 μM) MilliQ dH_2_0.

#### Data analysis

Standard methods in the LIMMA package [26] in R , available from the Bioconductor  were employed for preprocessing and normalization. Within-array normalization was first conducted by subtracting the median from the log-ratios for each array. A standard loess-normalization was then performed, where smoothing was based only on spots with abs(log-ratio) < 2.0 to avoid biases due to extreme skewness in the log-ratio distribution. For the determination of present and divergent genes, a new method that predicts sequence identity based on array signals was used, as described by Snipen et al. [27]. A brief description of the method follows: Initially, all array probe sequences were queried against the fully sequenced V583 reference genome (R-genome) in a BLAST search. The value *Rb *for each probe was defined as the number of identical bases in the best local alignment found by blastn, divided by the probe length. Given the sequence identity *Rb *for each probe, the corresponding array signal *Ra *will in general correlate in a positive way, *i.e*. stronger sequence identity yields stronger array signal. This postulation also holds for the unsequenced sample genome (S-genome), where *Sa *denote the array signal and *Sb *the unknown sequence identity. The basic idea is that *Sb *can be predicted from *Sa *based on how sequence identity *Rb *and array signal *Ra *relate to each other. A threshold of 0.75 was assigned to the *Sb*-values in order to obtain a categorical response of presence or divergence, *i. e*. genes with *Sb*-value > 0.75 were classified as present, while genes with *Sb*-value < 0.75 were classified as divergent. In a comparison with other methods for analyzing CGH data as reviewed by Carter et al. [28], the proposed method gave significantly better classification of present/divergent. Based on the fully sequenced genomes of V583 and OG1RF [15,29], tests gave sensitivity of 0.99 and specificity of 0.95 for detecting present probes based on CGH data.

#### Comparative phylogenomics

The relationship of the reference strain and the test strains based on the presence and divergence of genes was determined with Bayesian-based algorithms implemented through MR BAYES 3.1 [30], as previously described [31]. With samples and saves from every 40^th ^tree, 1.1 × 10^6 ^generations of four incrementally heated Markov chain Monte Carlos (MCMC) were performed on the CGH data by using an annealing temperature of 0.5, a burn-in of 100 000 MCMC generations and an 8-category distribution. Consensus trees and clade credibility values were visualized by using TreeView version 1.6.6 .

#### Microarray data accession number

The microarray data have been deposited in the ArrayExpress database with the series accession number E-TABM-466.

## Results

### MLST – allelic variation in community-derived fecal *E. faecalis *baby isolates

The MLST analysis was performed on 31 *E. faecalis*-isolates, obtained from 11 healthy Norwegian infants during their first year of life. These isolates were considered as legitimate representatives of commensal *E. faecalis *as they have been resident in the gut without causing any apparent negative effect to the health of the host. Infants A-C were sampled once each month during the first six months and once after 12 months, and a total of 8, 5 and 5 different isolates were recovered from the respective infants over the period of sampling. Infants D-K were sampled once during the first six months of life and 1–3 different isolates were obtained from these infants. From the 31 isolates, 12 different sequence types (STs) were identified, of which four were novel STs (ST161, ST162, ST163 and ST194; Table 1). Of these novel STs, ST161 and ST162, were single-locus variants (SLV; differing from the ancestor ST in one allele) of ST64 and ST72, respectively. ST64 and ST161 belong to the previously defined clonal complex CC8. In addition to the ST161 (n = 1) isolate that was detected in one of the infants, three different isolates displaying ST64 (n = 3) were obtained from the same infant (A) during the same month. Other clonal complexes that were represented within our collection of isolates were CC2 (n = 2), CC30 (n = 6), CC44 (n = 6) and CC55 (n = 1). The remaining isolates were singletons.

**Figure 1:**
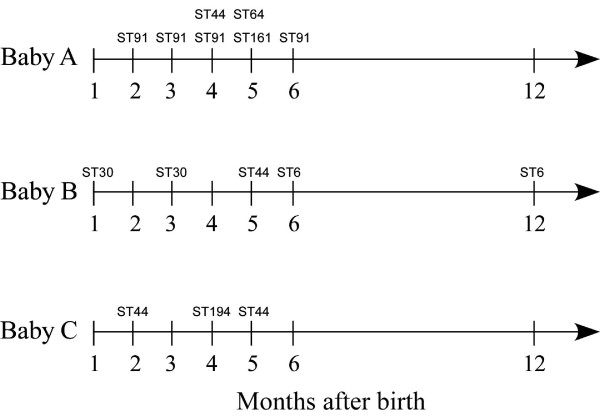
**The different sequence types that were detected in infants A-C during their first year of life**. ST; sequence type.

Several STs were detected multiple times, within different infants: ST30 was found in 5 of the 11 infants, while ST44 occurred in three different infants, and ST16 and ST162 were both detected in two different infants. The number of different STs for the three infants (A-C) monitored over 12 months, ranged between 2 and 4. Some of these STs were detected only for a short period of time, while other STs persisted throughout the sampling period (Figure 1). No *E. faecalis *isolates were obtained from infant A and C at 12 months of age. This observation was probably due to the conditions for enterococcal selection not being stringent enough.

### Distribution of virulence genes, bacteriocin genes and antibiotic resistance profiles

Single-concentration plate assays were used to assess resistance to ampicillin, chloramphenicol, erythromycin, gentamicin, tetracyline and vancomycin (see Table 1 and Additional file 2). Tetracycline resistance was the most prevalent resistance trait among the baby isolates (17/31). Moreover, a few of the isolates (n = 4) showed erythromycin- or high-level gentamicin resistance. All isolates were also examined for the presence of the putative virulence factors *ace*, *agg*, *cylL *and *esp *by means of PCR. *ace *was amplified from all isolates, while 27/31 isolates were *agg*^+^. *esp *is known to be associated with the *E. faecalis *pathogenicity island (PAI) [25], and was detected in 20/31 of the isolates. Cytolysin has been shown to contribute to virulence in animal models of enterococcal infections, and *cylL*, encoding one of the structural subunits of enterococcal cytolysin was present in 16/31 of the isolates by PCR. Cytolysin production was detected in 9/31 of the isolates on blood agar plates. The isolates were also examined for gelatinase activity, which have been associated with virulence (reviewed in [2]). 15/31 isolates were gelatinase positive (GelE^+^). Since the absence of the regulator (*fsrB*) can cause a lack of the gelatinase phenotype despite the presence of a positive *gelE *genotype [23], the isolates were also tested for the presence of *gelE *and *fsrB *by PCR. 29 of the isolates were *gelE*^+ ^and 18 were *fsrB*^+ ^(see Table 1 and Additional file 2). PCR-screening for content of bacteriocin genes among the test strains further discriminated between isolates with matching resistance profiles and virulence characteristics (see Additional file 2).

### Comparative genomic hybridization analysis

**Figure 2:**
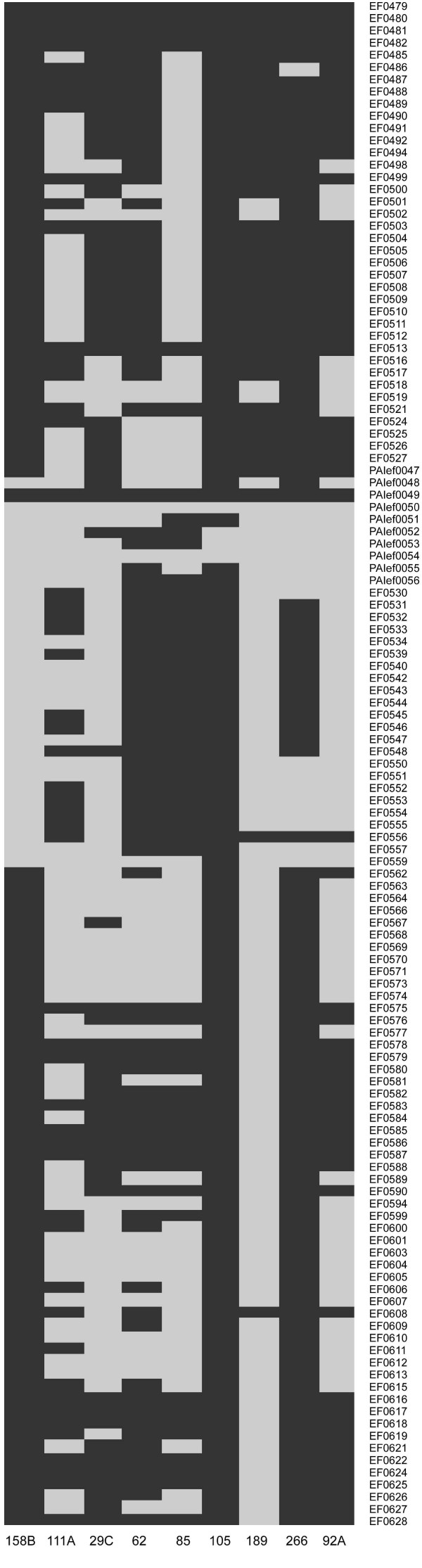
**Presence and divergence of PAI genes (123 of 129 open reading frames represented on the microarray) in nine *E. faecalis *baby isolates, as detected by CGH**. Genes PAIef**** correspond to EF**** genes in the PAI of strain MMH594 [25]. Putative enterococcal virulence genes located on the PAI include aggregation substance (*agg*; EF0485), cytolysin (*cyl*; EF0523–27 + PAIef0047–49) and enterococcal surface protein (*esp*; PAIef0056). Dark gray = present, light gray = divergent.

Whole-genome CGH experiments on *E. faecalis *have previously shown that the main variations between the sequenced reference strain V583 and test strains relate to regions coding for integrated phages, plasmids and transposable elements in V583 [17,18]. In our experiments, 169 genes were classified as divergent in all 9 isolates, 121 of which are chromosomal genes in V583 (see Additional file 3). The majority of the divergent chromosomal genes are located within the following 6 previously identified mobile genetic elements (MGE) in V583: efaC2 (EF0127–66; n = 19), phage01 (EF0303–55; n = 9), phage03 (EF1417–89; n = 16), *vanB *resistance region as defined by [17] (EF2284–2334; n = 48), efaC1 (EF2512–46; n = 17) and phage07 (EF2936–55; n = 6). A large fraction of the 121 chromosomal genes code for hypothetical proteins (n = 70) or conserved hypothetical proteins/conserved domain proteins (n = 35). Apparently, a great proportion of the variable gene pool consists of hypothetical ORFs and this seems to be a common trait among prokaryotes [32]. None of the MGEs were entirely divergent in all of the commensal isolates, *e.g*. the content of PAI genes in the isolates varied from 36 to 118 present genes of the 123 PAI ORFs represented on the array (Figure 2), and similar patterns of present and divergent genes between isolates may suggest that MGE genes are often transferred in modules. The fact that only 48 of the plasmid-encoded genes on the array (n = 167; pTEF1, pTEF2 and pTEF3) were divergent in all the baby isolates is consistent with such an evolutionary scenario; however, the isolates have not been tested for plasmid content to further explore this hypothesis. The results of the microarray analysis were generally consistent with the phenotypic tests and the PCR analysis of the presence of virulence-associated genes, with a few exceptions only: the isolate 111A was *esp*^+ ^by PCR, but *esp*^- ^by CGH. The same isolate was also *cylL*^+ ^by PCR and Cyl^+^, but the entire cytolysin locus, except from PAIef0049, was divergent by CGH. The Cyl^+ ^phenotypes of the isolates 158B and 189 were also inconsistent with the CGH data for some of the additional PAI genes on the array (PAIef0047–49). The observed inconsistencies between phenotypes/PCR and CGH may be due to sequence variations in the microarray-probe target sequences in the test stains.

Each of the fecal baby isolates showed a minimum of 76.5% presence of probes represented on the array (76.6% of the V583 genome). The CGH analysis classified 2092 of the 3093 chromosomal V583 ORFs as present in all 9 isolates. This set of shared genes is slightly higher than the core genomes that were previously reported for *E. faecalis *[17,18], probably due to the constricted environment and the limited geographical area, from which the isolates were obtained. The observed genetic variation among the investigated commensal *E. faecalis *shows that the genetic diversity is comparable to that among strains from other sources (food, clinical, environmental, animal husbandry etc) [17,18]. Our data confirmed the establishment of phage02 as a part of the *E. faecalis *core genome.

The *E. faecalis *V583 genome contains 35 probable phosphoenolpyruvate-dependent sugar phosphotransferase (PTS) systems and pathways for exploitation of 15 different sugars have been predicted [15]. Carbohydrate fermentation patterns obtained with API 50 CH kits for selected baby isolates whose gene content were also analyzed by CGH (see Additional file 4), showed only small differences in the metabolic capabilities of the test strains. Also, compared to API 50 CH results previously obtained for V583 [18], only minor variations were observed (see Additional file 4). These observations were supported by the high degree of conservation of genes encoding PTS components revealed by the CGH data. The recent publication of the *E. faecalis *OG1RF genome sequence revealed an *iol *operon that is not found in the V583 genome [29]. Interestingly, two of the baby isolates (111A and 105) were, according to the API assay, able to ferment myo-inositol. All the baby isolates were therefore surveyed for the presence of *iolE *and *iolR *by PCR. A total of 13 isolates, including 111A and 105, were both *iolE*^+ ^and *iolR*^+ ^(see Additional file 2). The presence of a partial *iol *operon in isolates 189 and 266 which were unable to ferment myo-inositol by the API assay is consistent with previous findings [29].

**Figure 3:**
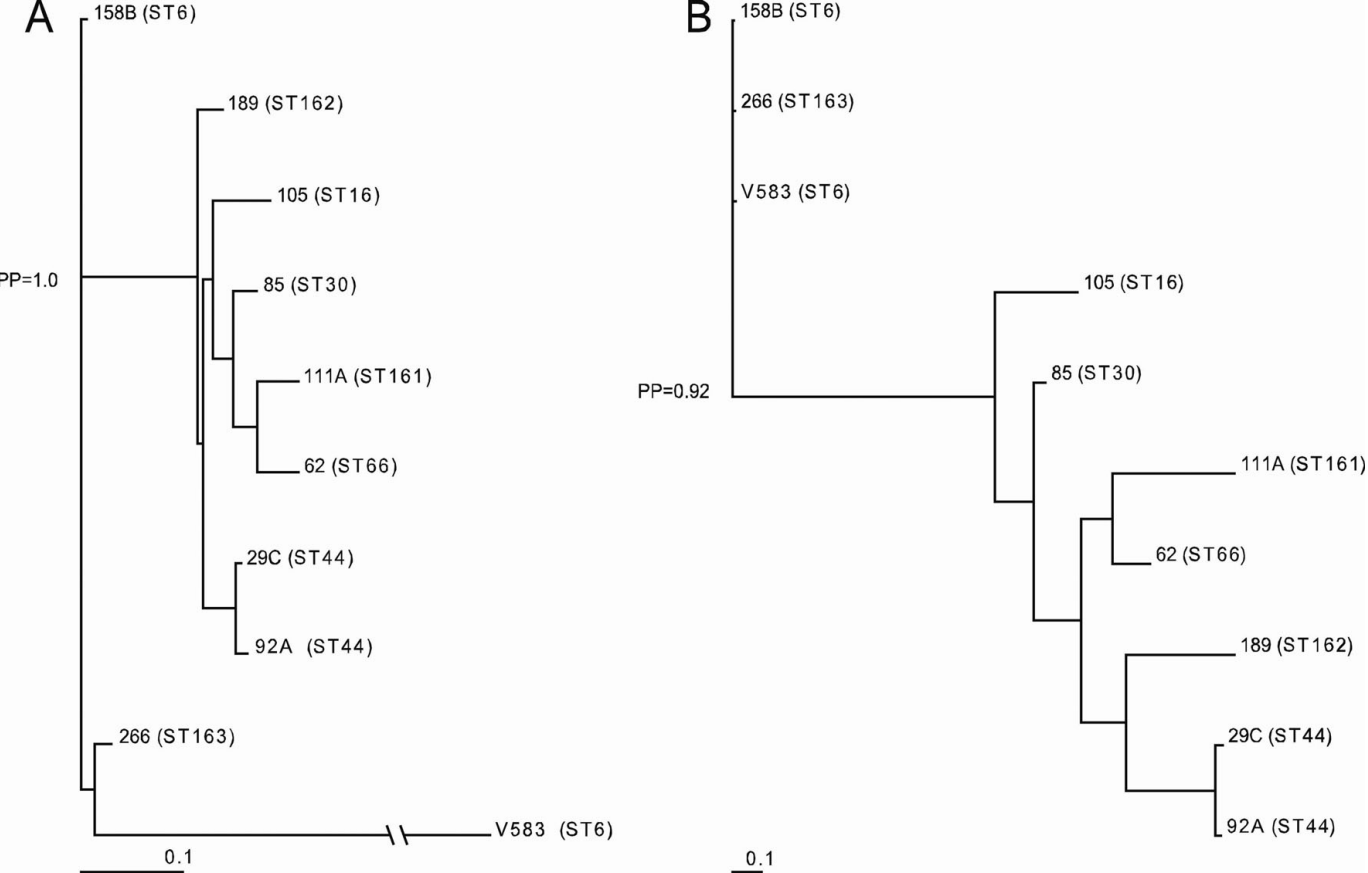
**Phylogenomic relationship of community-derived fecal baby isolates based on (A) total microarray probe set and (B) core variable (CV) genes, as detected by CGH**. Isolate names and sequence type (ST) are indicated at the end of the branches. Numerical values represent the posterior probability (*PP*) of support for internal branches.

Phylogenomic analyses of the CGH data using Bayesian-based algorithms revealed a distinct clade containing seven of the nine community-derived baby isolates (Bayesian posterior probabilities [*PP*] = 1.0; Figure 3A). Initial branching within this clade was also fairly reliable (*PP *> 0.80). The remaining three isolates seemed to be more divergent, and V583 formed a distinct out-group. Based on this analysis, isolate 266 (ST163) appeared more closely related to V583 (ST6) than isolate 158B, which also displays ST6. Nevertheless, the Bayesian phylogeny suggested that the lineages defined by CGH generally correlated with those identified by MLST: Within the latter clade, the isolates 92A and 29C (ST44) formed one separate clade (Figure 3A). To further examine the correlation between MLST and CGH, additional CGH data obtained from the literature, in addition to unpublished data from five additional non-baby isolates of *E. faecalis *were included in the analysis. In this extended analysis, only genes (n = 3043) represented on all three arrays that were used in the different studies were considered. The clustering of identical STs was here further supported (*PP *> 0.55; see Additional file 5). Previous studies have suggested that genomotyping of *E. faecalis *by CGH is heavily influenced by extensive horizontal transfer of MGEs in *E. faecalis *[17]. To further analyze the effect of MGEs in our data set, the CGH data were reanalyzed after all core- and MGE genes had been removed from the gene list, as previously described [33]. The MGE genes comprised putative MGEs predicted in V583 [15], as well as an additional phage-related region identified by McBride et al. [17]. This revision left a list of 370 genes (see Additional file 6). Lindsay and coworkers [33,34] previously denominated such genes core variable (CV; all genes minus core genes minus MGE genes). The phylogenetic tree based on the content of CV genes in the isolates recovered a clade containing the same seven baby isolates as in the analysis with the entire probe set (*PP *= 0.92; Figure 3B).

Of the 370 CV genes, 145 genes code for hypothetical proteins. Among the remaining functionally annotated CV genes, many genes are predicted to code for surface-exposed proteins in *E. faecalis*, *e.g*. the *cps *locus coding for the capsule in *E. faecalis *[35]. The *cps *locus of *E. faecalis *consists of 11 genes (*cpsABCDEFGHIJK*) and insertional inactivation of genes in this locus have resulted in mutants with enhanced susceptibility to phagocytic killing [35]. Three different *cps *polymorphisms have been identified in *E. faecalis *so far: 1) type 2 (*cps2*) which includes all 11 genes, 2) type 5 (*cps5*) which includes all genes except *cpsF *and 3) type 1 (*cps1*) with only *cpsA *and *cpsB *present. All three polymorphisms were detected among our test strains, by CGH (Table 1). The *cps *type has previously been found to be invariant within MLST sequence types in *E. faecalis *[17], and our data is consistent with this finding. Analysis of the CGH data with respect to the *cps *type suggested that the *E. faecalis *PAI, or segments thereof, may be enriched among *cps2*-isolates. These observations support the hypothesis of convergence of enterococcal virulence determinants and *cps2 *by independent selection in *E. faecalis *[17].

## Discussion

Enterococci are among the first bacteria to colonize the neonatal GI tract [1]. Though originally considered as harmless commensals, the enterococci now rank among the leading causes of nosocomial infections [36,37]. The present study was undertaken in an attempt to further explore the differences in the genetic make-up of *E. faecalis*. A total of 31 community-derived fecal baby isolates were sequence typed by MLST and characterized with respect to antibiotic resistance and properties associated with virulence. A subset of the isolates was genomotyped using genome-wide DNA microarrays.

By MLST analysis, the 31 baby isolates were resolved into 12 STs and grouped into 11 genetic lineages, including 6 major clonal complexes (CCs) and 5 singletons . Analyses with the MLST scheme employed in the present study have previously defined distinct clonal complexes of *E. faecalis *associated with the hospital environment, so-called high-risk enterococcal clonal complexes (HiRECC; CC2, CC9, CC40 and CC87) [20,38]. Of the isolates included in this study, only 158B and 226B (ST6) grouped into one of these complexes (CC2). These isolates were obtained towards the end of the sampling period, and may therefore have been introduced through habituation to solid food or from the environment through fecal-oral contamination. To our knowledge, none of the infants were admitted to the hospital during the period of sampling, however, hospital contact cannot be excluded as a source for ST6 isolates.

According to our results, several of the putative enterococcal virulence factors were widespread among the commensal baby isolates. These findings are in line with previous reports [11], and may reflect the adaptive functions that these factors can hold in non-virulent contexts, as indicated by Semedo et al. [11]. Several of the virulence traits and antibiotic resistant phenotypes were common for all the isolates within the clonal complexes; however, the strain set is too small to deduce statistically significant association of features with clonal identity of isolates.

Overall, our results highlight the importance of phenotypic assays to confirm genomics data as revealed by PCR and CGH. PCR confirmed the presence of *cylL *in the eight isolates that displayed hemolytic activity in our study, however, *cylL *was also found to be present in an additional eight Cyl^-^-isolates. A similar discrepancy between fermentation capabilities and the presence of *iolE *and *iolR *was also observed. Moreover, three isolates carrying both *gelE *and *fsrB *came out as GelE^- ^in the plate assay. The confined genotype-phenotype correlation that is here reported, visualizes the need for phenotypic confirmation of genotypes.

*esp *which is known to be associated with the *E. faecalis *pathogenicity island (PAI) [25], was detected in two thirds of the commensal isolates by PCR. According to the CGH data, none of the baby isolates contained complete PAIs. These findings were as expected, considering that the PAI has been shown to be enriched among infection-derived enterococcal isolates [25]. More surprisingly, but consistent with a previous report [39], all the isolates studied contained some PAI genes. Several of the baby isolates showed similar patterns of present and divergent PAI genes (Figure 2). This suggests that the evolution of the enterococcal PAI may be driven by insertion and deletion of larger modules, as hypothesized in [39]. Shankar et al. also suggested that parts of the enterococcal PAI originate from pheromone-responsive plasmids, with subsequent indels of transposable elements driving the evolution of the PAI [39]. Indeed, conjugal transfer of a segment of the *E. faecalis *PAI has been demonstrated [40]. The CGH revealed a high degree of plasticity within all the MGEs represented on the microarray. These "mosaic structures" may reflect a complex evolutionary history of elements that have been frequently rearranged by horizontal gene transfer (HGT) and homologous recombination.

According to the CGH data presented here, a preliminary *E. faecalis *core genome consisting of 2092 (out of the 3093 chromosomal V583 ORFs) can be delineated. Compiled analysis of the data from Aakra et al. and McBride et al. [17,18] with the data from the present study produced a core genome estimate of 1722 genes. An additional 62 genes were only represented on one or two of the three different arrays used, but were defined as core genes in these experiments. Although the size of the core genome may fluctuate due to the stringency of the statistical methods used in the different studies, our data do add substantial information on the *E. faecalis *core genome.

In general, the genomic variation between isolates that are evolutionary -linked, *e.g*. isolates with the same ST, was expected to be lower than the variation between isolates that belong to different evolutionary lineages. Bayesian-based phylogenetic analysis confirmed these expectations (Figure 3A). McBride et al. previously reported genomotyping by CGH to be biased by the activity of MGEs in *E. faecalis *[17], and we therefore repeated the Bayesian analysis, using the CV genes, only. The phylogenetic analysis based on CV genes recovered the same patterns of relatedness as the analysis comprising all genes, with slight internal rearrangements of branches (Figure 3B). These rearrangements supported the hypothesis on the distribution of mobile elements as a source of genomic diversity in *E. faecalis*. Moreover, our data suggest that within lineages, most of the variation detected by CGH is due to MGEs. However, the conserved clade identified by the analyses based both on the CV genes and the complete gene-set, indicates that also other and more complex discriminatory factors contribute to the genomic diversity in *E. faecalis*. Since an overall correlation between CGH and MLST was revealed, it is reasonable to believe that genes contributing to the formation of clades, *i.e*. lineage-specific genes may be identified. In the 7 baby isolates that formed a clade in the phylogenetic analysis, we were able to recognize 137 genes that were divergent, but present in the remaining three isolates (including the reference strain). The majority of these genes were MGE genes located in phage03 (n = 39), phage06 (n = 28) and a phage-related region identified by McBride et al. [17] (EF2240–82/EF2335–51; n = 44). Lepage et al. have previously reported phage03 to be absent from several food isolates [16]. Since ST6 is part of CC2, which has been found to be significantly enriched among nosocomial isolates, phage03 may potentially represent an element associated with increased fitness in the hospital environment. The latter report also identified eight genes as potential markers for the V583/MMH594-lineage [16]. V583 and MMH594 both display ST6 [17], and five of the eight genes (EF2250, EF2253, EF2254, EF3155 and EF3252) were also present in the ST6-isolates (158B and LMGT3303; results not shown) analyzed by CGH in our study.

Comparative genome analyses have revealed that pathogen evolution often progresses through gene acquisition via HGT [32]. The 169 genes that were characterized as divergent in all the community-derived baby isolates by CGH may be potential pathogen-specific genes in *E. faecalis*, or genes that are specific to V583. However, additional CGH data from both pathogenic and non-pathogenic isolates are needed to address this issue. Vancomycin-resistant *E. faecalis *(VREfs) isolates appear to be widely spread in hospital environments, while isolation of VREfs from healthy volunteers rarely occurs [41-44]. Accordingly, the *vanB *operon was divergent in all the isolates studied by CGH in our lab (altogether 21 strains;[18] and results not shown). In addition to gene acquisition, pathoadaptive mutations via gene loss also plays an important role in evolution of bacteria [45]. A disadvantage of the comparative genomic analyses presented here, is that the comparison of gene content is based on a single reference strain (V583), only. The *E. faecalis *OG1RF genome showed that, in addition to a shared core of 2474 ORFs [29], both the V583 and the OG1RF genome carry unique genes, suggesting that the *E. faecalis *pan-genome will be further extended as more strains will be sequenced. The availability of additional *E. faecalis *genome sequences and the construction of a pan-species array would further increase the sensitivity of such approaches.

## Conclusion

The data presented here suggest that the genetic variation among the investigated commensal *E. faecalis *is comparable to the genetic variation previously detected in a strain set thought to be representative of the major *E. faecalis *lineages. The widespread distribution of putative virulence determinants in the fecal baby isolates in this study supports the conception of enterococcal virulence, not as a result of any single virulence factor, but as a more complex process. Population structure studies of *E. faecalis *by MLST have identified so-called high-risk enterococcal clonal complexes (HiRECCs), defined as distinct genetic complexes that predominate among nosocomial infections. The failure to identify a shared set of pathogen-specific genes in *E. faecalis *so far opens up the possibility that the fitness and virulence of different HIRECCs may be due to genes that are unique within a lineage, but that the combined effects of the different gene-sets result in the same phenotype, *i.e*. infection. The observed correlation between CGH and MLST presented here, may offer a method for the identification of lineage-specific genes, and may therefore add clues on how to distinguish pathogenic from commensal *E. faecalis*.

## Authors' contributions

MS participated in the design of the study, carried out the experimental work and drafted the manuscript. ÅAa participated in the design of the study and helped to draft the manuscript. LS proposed the statistical analysis and did the programming and statistical analysis in R. DAB and IFN participated in the design of the study and assisted in critical review of the manuscript. All authors read and approved the final manuscript.

## Supplementary Material

Additional File 1 **Primers used in this study**. A table of primers used in this study. Click here for file

Additional File 2 **Genotypic and phenotypic characteristics of *E. faecalis *baby isolates**. A table of the results from the phenotypic- and genotypic assays conducted in this study. Click here for file

Additional File 3 **Genes divergent in all the baby isolates**. A table of genes that were classified as divergent in all the baby isolates analyzed by CGH. Click here for file

Additional File 4 **Fermentation patterns from API 50 CH assays**. A table of results from fermentation profiling of selected *E. faecalis *baby isolates by API 50 CH. Click here for file

Additional File 5 **The phylogenomic relationship of *E. faecalis *isolates based on gene content, as detected by CGH**. A phylogenetic tree based on CGH data from the present study, in addition to previously published CGH data from the literature. Click here for file

Additional File 6 **Core variable genes**. A table of genes that were classified as core variable by CGH. Click here for file
