# Supplementary material for: Comparative genomics of Enterococcus faecalis from healthy Norwegian infants
Source: BMC Genomics. 2009 Apr 24;10:194. doi: 10.1186/1471-2164-10-194 (PMC2680900; doi:10.1186/1471-2164-10-194)
Supplement: Additional File 3 — Genes divergent in all the baby isolates. A table of genes that were classified as divergent in all the baby isolates analyzed by CGH. [file 1471-2164-10-194-S3.doc]

**Additional file 3.** **Genes that were divergent in all baby isolates analyzed by CGH**.

| ORF | Gene product |
| --- | --- |
| EF0127 | Conserved hypothetical protein |
| EF0128 | Hypothetical protein |
| EF0129 | Transcriptional regulator, Cro/CI family |
| EF0130 | Hypothetical protein |
| EF0132 | Hypothetical protein |
| EF0133 | Hypothetical protein |
| EF0134 | Hypothetical protein |
| EF0136 | Hypothetical protein |
| EF0137 | Nucleotidyltransferase domain protein |
| EF0138 | Conserved domain protein |
| EF0142 | Conserved hypothetical protein |
| EF0143 | Transcriptional regulator, Cro/CI family |
| EF0144 | Conserved domain protein |
| EF0146 | Surface exclusion protein, putative |
| EF0147 | Hypothetical protein |
| EF0149 | Aggregation substance, putative |
| EF0151 | Hypothetical protein |
| EF0161 | Hypothetical protein |
| EF0164 | Lipoprotein, putative |
| EF0313 | Hypothetical protein |
| EF0316 | Hypothetical protein |
| EF0318 | Conserved hypothetical protein |
| EF0326 | Conserved hypothetical protein |
| EF0327 | Hypothetical protein |
| EF0330 | SNF2 domain protein |
| EF0352 | Hypothetical protein |
| EF0354 | Holin, putative |
| EF0355 | Endolysin, putative |
| EF1420 | Hypothetical protein |
| EF1421 | Conserved hypothetical protein |
| EF1431 | Hypothetical protein |
| EF1432 | Hypothetical protein |
| EF1437 | Hypothetical protein |
| EF1438 | Hypothetical protein |
| EF1439 | Hypothetical protein |
| EF1440 | Conserved hypothetical protein TIGR01671 |
| EF1442 | DNA topoisomerase domain protein |
| EF1452 | Adenine methyltransferase, putative |
| EF1453 | Conserved hypothetical protein |
| EF1454 | Terminase, small subunit, internal deletion |
| EF1468 | Conserved hypothetical protein |
| EF1487 | Hypothetical protein |
| EF1488 | Hypothetical protein |
| EF1489 | Hypothetical protein |
| EF2085 | Conserved hypothetical protein |
| EF2118 | Conserved domain protein |
| EF2119 | Hypothetical protein |
| EF2142 | Transcriptional regulator, Cro/CI family |
| EF2143 | Conserved hypothetical protein |
| EF2144 | Lipoprotein, putative |
| EF2283 | Site-specific recombinase, resolvase family, putative |
| EF2284 | Hypothetical protein |
| EF2285 | Hypothetical protein |
| EF2286 | ParB-like nuclease domain protein |
| EF2287 | Hypothetical protein |
| EF2288 | Hypothetical protein |
| EF2289 | Hypothetical protein |
| EF2290 | RNA polymerase sigma-70 factor, ECF subfamily |
| EF2291 | Transcriptional regulator, Cro/CI family |
| EF2292 | Hypothetical protein |
| EF2293 | D-alanyl-D-alanine dipeptidase |
| EF2294 | D-alanine--D-lactate ligase |
| EF2295 | D-specific alpha-keto acid dehydrogenase |
| EF2296 | Vancomycin B-type resistance protein VanW |
| EF2297 | D-alanyl-D-alanine carboxypeptidase |
| EF2298 | Sensor histidine kinase VanSB |
| EF2299 | DNA-binding response regulator VanRB |
| EF2300 | Streptomycin resistance protein, putative |
| EF2302 | Conserved hypothetical protein |
| EF2303 | Conserved hypothetical protein |
| EF2304 | Transcriptional regulator, Cro/CI family |
| EF2305 | Toprim domain protein |
| EF2306 | Conserved hypothetical protein |
| EF2308 | Hypothetical protein |
| EF2309 | Hypothetical protein |
| EF2310 | Hypothetical protein |
| EF2311 | Hypothetical protein |
| EF2312 | DNA topoisomerase III |
| EF2313 | Hypothetical protein |
| EF2314 | Bacteriocin, putative |
| EF2315 | Hypothetical protein |
| EF2316 | Conserved domain protein |
| EF2317 | Hypothetical protein |
| EF2318 | Peptidase, M23/M37 family |
| EF2319 | Hypothetical protein |
| EF2320 | TraE protein, putative |
| EF2321 | Hypothetical protein |
| EF2322 | Conserved domain protein |
| EF2324 | Modification methylase MUNI putative, truncation |
| EF2325 | Hypothetical protein |
| EF2327 | Hypothetical protein |
| EF2328 | TraG family protein |
| EF2329 | Hypothetical protein |
| EF2330 | Hypothetical protein |
| EF2331 | Hypothetical protein |
| EF2332 | Conserved domain protein |
| EF2333 | Hypothetical protein |
| EF2334 | Conserved domain protein |
| EF2512 | Lipoprotein, putative |
| EF2517 | Conjugal transfer protein, putative |
| EF2518 | Conserved domain protein |
| EF2519 | Conserved hypothetical protein |
| EF2520 | Conserved hypothetical protein |
| EF2522 | Hypothetical protein |
| EF2526 | Hypothetical protein |
| EF2527 | Conserved domain protein |
| EF2528 | Transcriptional regulator, Cro/CI family |
| EF2529 | Conserved hypothetical protein |
| EF2530 | Hypothetical protein |
| EF2532 | Hypothetical protein |
| EF2533 | FtsK/SpoIIIE family protein |
| EF2537 | Hypothetical protein |
| EF2538 | Hypothetical protein |
| EF2539 | Hypothetical protein |
| EF2540 | Hypothetical protein |
| EF2938 | Conserved hypothetical protein |
| EF2939 | Cold-shock domain family protein |
| EF2943 | Hypothetical protein |
| EF2947 | Conserved domain protein |
| EF2949 | Hypothetical protein |
| EF2950 | Hypothetical protein |
| EFA0001 | Replication-associated protein RepA |
| EFA0002 | Pheromone shutdown protein TraB |
| EFA0003 | TraC protein |
| EFA0004 | TraA protein |
| EFA0010 | Multidrug resistance protein |
| EFA0012 | Replication protein |
| EFA0014 | Drug resistance transporter, putative |
| EFA0015 | Hypothetical protein |
| EFA0016 | Transposase, IS6 family |
| EFA0017 | Hypothetical protein |
| EFA0021 | Conserved domain protein |
| EFA0025 | Hypothetical protein |
| EFA0026 | Hypothetical protein |
| EFA0029 | Hypothetical protein |
| EFA0030 | Conserved hypothetical protein |
| EFA0054 | Regulatory protein TraE1 |
| EFA0058 | RepE protein |
| EFA0065 | Conserved hypothetical protein |
| EFA0067 | PTS system, IIABC components |
| EFA0069 | Sucrose-6-phosphate hydrolase |
| EFA0070 | Sucrose operon repressor ScrR |
| EFA0071 | PemK family protein |
| EFA0081 | Conserved hypothetical protein |
| EFA0082 | Replication-associated protein RepC |
| EFA0083 | Replication-associated protein RepB |
| EFB0003 | Pheromone shutdown protein TraB |
| EFB0004 | TraC protein |
| EFB0005 | Transcriptional regulator, Cro/CI family |
| EFB0005.1 | Hypothetical protein |
| EFB0007 | Probable pheromone-responsive regulatory protein R |
| EFB0031 | Hypothetical protein |
| EFB0033 | Conserved hypothetical protein |
| EFB0034 | Conserved domain protein |
| EFB0050 | Toxin ABC transporter, ATP-binding/permease protein |
| EFB0053 | Conserved domain protein |
| EFB0054 | Conserved hypothetical protein |
| EFB0055 | Lipoprotein, putative |
| EFB0059 | Hypothetical protein |
| EFB0060 | ImpB/MucB/SamB family protein |
| EFB0061 | Hypothetical protein |
| EFC0005 | Restriction endonuclease related protein |
| EFC0008 | Conserved domain protein |
| EFC0013 | Hypothetical protein |
| EFC0014 | Hypothetical protein |
| EFC0016 | Hypothetical protein |
| EFC0017 | Hypothetical protein |
| PAIef0050 | Hypothetical protein |
| PAIef0054 | Hypothetical protein |
