# Supplementary figures and images for: Comparative genomics of Enterococcus faecalis from healthy Norwegian infants
Source: BMC Genomics. 2009 Apr 24;10:194. doi: 10.1186/1471-2164-10-194 (PMC2680900; doi:10.1186/1471-2164-10-194)

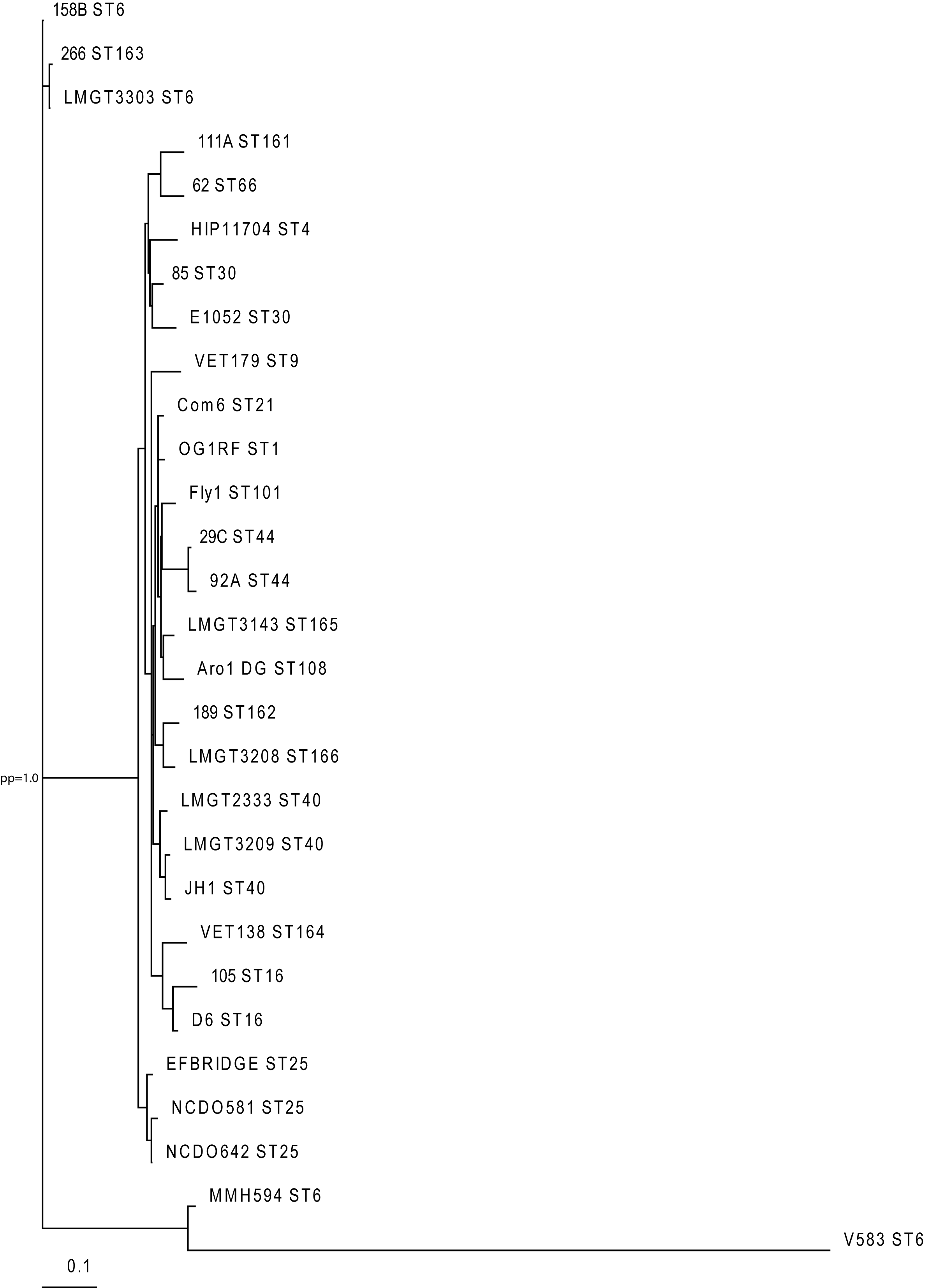

Supplement: Additional File 5 — The phylogenomic relationship of E. faecalis isolates based on gene content, as detected by CGH. A phylogenetic tree based on CGH data from the present study, in addition to previously published CGH data from the literature. [file 1471-2164-10-194-S5.png]
