# Supplementary material for: Comparative genomics of Enterococcus faecalis from healthy Norwegian infants
Source: BMC Genomics. 2009 Apr 24;10:194. doi: 10.1186/1471-2164-10-194 (PMC2680900; doi:10.1186/1471-2164-10-194)
Supplement: Additional File 6 — Core variable genes. A table of genes that were classified as core variable by CGH. [file 1471-2164-10-194-S6.doc]

**Additional file 6.** **Core variable genes as detected by CGH.**

| ORF | Gene product |
| --- | --- |
| EF0011 | DHH family protein |
| EF0052 | Hypothetical protein |
| EF0053 | DNA polymerase III, epsilon subunit |
| EF0054 | Hypothetical protein |
| EF0063 | Pheromone binding protein, putative |
| EF0066 | Holliday junction DNA helicase RuvA |
| EF0068 | Hypothetical protein |
| EF0086 | Conserved domain protein |
| EF0092 | Hypothetical protein |
| EF0093 | Cell wall surface anchor family protein |
| EF0112 | Conserved domain protein |
| EF0113 | Hypothetical protein |
| EF0115 | Endoribonuclease L-PSP, putative |
| EF0117 | Transcriptional regulator, GntR family |
| EF0118 | Ornithine cyclodeaminase, putative |
| EF0119 | Phenazine biosynthesis protein PhzF family |
| EF0122 | Conserved domain protein |
| EF0123 | Conserved hypothetical protein |
| EF0124 | Hypothetical protein |
| EF0125 | IS256, transposase |
| EF0126 | Conserved hypothetical protein |
| EF0183 | Hypothetical protein |
| EF0184 | Hypothetical protein |
| EF0239 | Cobalt transport family protein |
| EF0256 | Peptidyl-tRNA hydrolase |
| EF0263 | tRNA(Ile)-lysidine synthetase |
| EF0277 | Methylated-DNA--protein-cysteine S-methyltransferase |
| EF0281 | Hypothetical protein |
| EF0356 | Hypothetical protein |
| EF0360 | SugE protein |
| EF0367 | Conserved hypothetical protein |
| EF0373 | Sensor histidine kinase |
| EF0376 | Hypothetical protein |
| EF0379 | Death-on-curing family protein |
| EF0380 | Conserved hypothetical protein |
| EF0392 | Hypothetical protein |
| EF0393 | Hypothetical protein |
| EF0395 | Methionine synthase, putative |
| EF0402 | Na+/H+ antiporter |
| EF0405 | Hydrolase, haloacid dehalogenase-like family |
| EF0419 | Conserved hypothetical protein |
| EF0428 | Conserved hypothetical protein |
| EF0429 | TRAP dicarboxylate transporter, DctP subunit |
| EF0430 | TRAP dicarboxylate transporter, DctQ subunit |
| EF0431 | TRAP dicarboxylate transporter, DctM subunit |
| EF0432 | Transcriptional regulator, AraC family |
| EF0433 | Rhamnulokinase, putative |
| EF0434 | L-rhamnose isomerase |
| EF0435 | Rhamnulose-1-phosphate aldolase |
| EF0436 | Conserved hypothetical protein |
| EF0437 | Transcriptional regulator, AraC family |
| EF0438 | Conserved domain protein |
| EF0441 | Hypothetical protein |
| EF0442 | Hypothetical protein |
| EF0468 | LemA family protein |
| EF0469 | Conserved domain protein |
| EF0642 | Hypothetical protein |
| EF0653 | Hypothetical protein |
| EF0657 | Transcriptional regulator, DeoR family |
| EF0685 | Rotamase family protein |
| EF0691 | Methyltransferase, putative |
| EF0696 | Tagatose 1,6-diphosphate aldolase |
| EF0708 | Conserved hypothetical protein |
| EF0723 | Hypothetical protein |
| EF0738 | Hypothetical protein |
| EF0751 | Conserved hypothetical protein |
| EF0752 | Conserved hypothetical protein |
| EF0753 | Hypothetical protein |
| EF0754 | Conserved hypothetical protein |
| EF0755 | Conserved hypothetical protein |
| EF0756 | Hypothetical protein |
| EF0759 | SapB protein, putative |
| EF0766 | Conserved hypothetical protein |
| EF0767 | Conserved hypothetical protein |
| EF0775 | Gram positive anchor protein, putative |
| EF0780 | MutT/nudix family protein |
| EF0802 | Hypothetical protein |
| EF0810 | Conserved hypothetical protein |
| EF0811 | Hypothetical protein |
| EF0812 | Glucuronyl hydrolase, putative |
| EF0813 | Glycosyl hydrolase, family 35 |
| EF0814 | Transcriptional regulator, GntR family |
| EF0815 | PTS system, IIAB components |
| EF0816 | PTS system, IIC component |
| EF0817 | PTS system, IID component |
| EF0818 | Polysaccharide lyase, family 8 |
| EF0823 | Hypothetical protein |
| EF0856 | Aldo/keto reductase family protein |
| EF0860 | Membrane protein, putative |
| EF0912 | Peptide ABC transporter, ATP-binding protein |
| EF0919 | Acetyltransferase, GNAT family |
| EF0931 | Hypothetical protein |
| EF0937 | Conserved hypothetical protein |
| EF0957 | Glycosyl hydrolase, family 65 |
| EF0959 | Hypothetical protein |
| EF0965 | Conserved hypothetical protein |
| EF0966 | Transcriptional regulator, MerR family |
| EF0967 | Conserved domain protein |
| EF0973 | Proline dipeptidase |
| EF0992 | Phospho-N-acetylmuramoyl-pentapeptide-transferase |
| EF1015 | Hypothetical protein |
| EF1029 | Conserved hypothetical protein |
| EF1031 | Phosphorylase family protein |
| EF1063 | Conserved hypothetical protein |
| EF1072 | Galactose operon repressor galR |
| EF1076 | Streptomycin 3-adenylyltransferase, putative |
| EF1082 | Hypothetical protein |
| EF1084 | Universal stress protein family |
| EF1095 | Hypothetical protein |
| EF1099 | Collagen adhesin protein |
| EF1100 | ABC transporter, ATP-binding/permease protein |
| EF1117 | Amino acid ABC transporter, permease protein |
| EF1127 | Putative transport protein SgaT protein |
| EF1136 | Hypothetical protein |
| EF1145 | Conserved hypothetical protein |
| EF1164 | HD domain protein |
| EF1172 | Teichoic acid biosynthesis protein B, putative |
| EF1173 | Glycosyl transferase, WecB/TagA/CpsF family |
| EF1174 | Hypothetical protein |
| EF1175 | Glycerol-3-phosphate cytidylyltransferase |
| EF1177 | Hypothetical protein |
| EF1179 | Fructokinase |
| EF1193 | DNA-binding response regulator VicR |
| EF1216 | Hypothetical protein |
| EF1217 | Lipoprotein, putative |
| EF1229 | Conserved hypothetical protein |
| EF1266 | Hypothetical protein |
| EF1300 | Cell division protein, FtsW/RodA/SpovE family |
| EF1311 | Conserved hypothetical protein |
| EF1321 | Permease domain protein |
| EF1329 | HesA/MoeB/ThiF family protein |
| EF1330 | Hypothetical protein |
| EF1331 | ABC transporter, ATP-binding protein |
| EF1332 | Membrane protein, putative |
| EF1333 | ABC transporter, ATP-binding protein |
| EF1334 | AgrC domain protein |
| EF1335 | Sensor histidine kinase, putative |
| EF1336 | Response regulator |
| EF1337 | Conserved hypothetical protein |
| EF1339 | Conserved hypothetical protein |
| EF1369 | Transcriptional regulator, Cro/CI family |
| EF1382 | Peptidase T |
| EF1392 | Molybdenum cofactor biosynthesis protein MoaC |
| EF1406 | Excinuclease ABC, subunit C |
| EF1407 | Hypothetical protein |
| EF1490 | Hypothetical protein |
| EF1507 | Hypothetical protein |
| EF1511 | Mandelate racemase/muconate lactonizing enzyme family protein |
| EF1545 | ATP-dependent DNA helicase RecQ |
| EF1565 | Prephenate dehydrogenase |
| EF1568 | Prephenate dehydratase |
| EF1582 | Transketolase, authentic frameshift |
| EF1589 | Acetyltransferase, GNAT family |
| EF1614 | DNA topoisomerase IV, A subunit |
| EF1619 | Carbon dioxide concentrating mechanism protein CcmL, putative |
| EF1620 | Hypothetical protein |
| EF1647 | Heat shock protein HslV |
| EF1664 | Conserved hypothetical protein |
| EF1668 | Transcriptional regulator, MarR family |
| EF1669 | Glyoxylase family protein |
| EF1670 | Phospholipase/carboxylesterase family protein |
| EF1672 | Permease protein, putative |
| EF1673 | ABC transporter, ATP-binding protein |
| EF1674 | Hypothetical protein |
| EF1685 | Hemolysin III |
| EF1689 | Hypothetical protein |
| EF1735 | Hypothetical protein |
| EF1768 | ABC transporter, ATP-binding protein |
| EF1783 | Phosphoribosylformylglycinamidine synthetase I |
| EF1812 | Hypothetical protein |
| EF1820 | Histidine kinase, putative |
| EF1821 | AgrBfs protein |
| EF1822 | Response regulator |
| EF1823 | N-acetylmuramoyl-L-alanine amidase, family 4 |
| EF1824 | Glycosyl hydrolase, family 31/fibronectin type III domain protein |
| EF1825 | Conserved domain protein |
| EF1826 | Alcohol dehydrogenase, zinc-containing |
| EF1827 | Conserved hypothetical protein |
| EF1828 | Glycerol uptake facilitator protein, putative |
| EF1829 | PTS system, IID component |
| EF1830 | PTS system, IIC component |
| EF1833 | PTS system component, authentic frameshift |
| EF1834 | Galactose-6-phosphate isomerase, LacB subunit |
| EF1835 | Galactose-6-phosphate isomerase, LacA subunit |
| EF1836 | PTS system, IIA component, putative |
| EF1837 | PTS system, IIB component, putative |
| EF1838 | PTS system, IIC component |
| EF1839 | Lactose phosphotransferase system repressor LacR |
| EF1841 | Lipoprotein, putative, authentic frameshift |
| EF1843 | Polysaccharide deacetylase family protein |
| EF1844 | Hypothetical protein |
| EF1846 | Hypothetical protein |
| EF1896 | Cell wall surface anchor family protein |
| EF1897 | Hypothetical protein |
| EF1900 | 16S rRNA processing protein RimM |
| EF1920 | C4-dicarboxylate anaerobic carrier |
| EF1921 | Inosine-uridine preferring nucleoside hydrolase |
| EF1922 | Transcriptional regulator, LacI family/carbohydrate kinase, PfkB family protein |
| EF1923 | Hypothetical protein |
| EF1929 | Glycerol kinase |
| EF1958 | Deoxyguanosinetriphosphate triphosphohydrolase, putative |
| EF1959 | Conserved domain protein |
| EF1979 | ATPase, AAA family |
| EF2063 | Transcriptional regulator, AraC family |
| EF2076 | Endocarditis specific antigen |
| EF2158 | Pyruvate ferredoxin/flavodoxin oxidoreductase family protein |
| EF2164 | Membrane protein, putative |
| EF2165 | NAD-dependent epimerase/dehydratase family protein |
| EF2166 | Membrane protein, putative |
| EF2167 | Glycosyl transferase, group 2 family protein |
| EF2168 | LicD1 protein, putative |
| EF2169 | Membrane protein, putative |
| EF2170 | Glycosyl transferase, group 2 family protein |
| EF2171 | Epimerase/dehydratase, putative |
| EF2172 | 2-C-methyl-D-erythritol 4-phosphate cytidylyltransferase |
| EF2173 | ISEf1, transposase |
| EF2174 | Conserved domain protein |
| EF2175 | LicD-related protein |
| EF2176 | Glycosyl transferase, group 2 family protein |
| EF2186 | ISEf1, transposase |
| EF2188 | IS256, transposase |
| EF2196 | Glycosyl transferase, group 2 family protein |
| EF2205 | Aminopeptidase pep, authentic frameshift |
| EF2211 | Conserved hypothetical protein TIGR01655 |
| EF2220 | Conserved hypothetical protein |
| EF2228 | Tryptophanyl-tRNA synthetase |
| EF2235 | Glucuronyl hydrolase, putative |
| EF2360 | Conserved hypothetical protein |
| EF2382 | Conserved domain protein |
| EF2385 | Hypothetical protein |
| EF2386 | ParB-like nuclease domain protein |
| EF2387 | Hypothetical protein |
| EF2388 | Hypothetical protein |
| EF2389 | Hypothetical protein |
| EF2411 | Diacylglycerol kinase |
| EF2449 | Competence protein comEA |
| EF2452 | Methylase, putative |
| EF2454 | Conserved hypothetical protein |
| EF2460 | GTP-binding protein TypA |
| EF2464 | Conserved domain protein |
| EF2465 | Hypothetical protein |
| EF2466 | Conserved domain protein |
| EF2467 | Conserved hypothetical protein |
| EF2479 | Conserved hypothetical protein |
| EF2480 | Conserved hypothetical protein |
| EF2485 | ABC transporter, permease protein |
| EF2486 | ABC transporter, ATP-binding protein |
| EF2487 | UDP-galactopyranose mutase |
| EF2488 | Lipoprotein, putative |
| EF2489 | MurB family protein |
| EF2490 | Conserved hypothetical protein |
| EF2491 | Glycosyl transferase, group 2 family protein |
| EF2492 | Glycosyl transferase, group 2 family protein |
| EF2493 | Teichoic acid biosynthesis protein, putative |
| EF2501 | Arsenate reductase, putative |
| EF2505 | Cell wall surface anchor family protein |
| EF2572 | Molybdenum transport domain protein |
| EF2574 | Endoribonuclease L-PSP, putative |
| EF2577 | Aspartate/ornithine carbamoyltransferase family protein |
| EF2584 | Hypothetical protein |
| EF2593 | ABC transporter, ATP-binding/permease protein |
| EF2595 | Guanylate kinase |
| EF2622 | Conserved hypothetical protein |
| EF2628 | N-acetylmuramoyl-L-alanine amidase, family 4 |
| EF2629 | Hypothetical protein |
| EF2630 | Transcriptional regulator |
| EF2647 | Permease, GntP family |
| EF2648 | Conserved hypothetical protein |
| EF2662 | Choline binding protein |
| EF2665 | RNA methyltransferase, TrmH family |
| EF2666 | Ribosomal RNA large subunit methyltransferase A, putative |
| EF2668 | Magnesium transporter |
| EF2677 | Negative regulator of genetic competence MecA, putative |
| EF2710 | Amino acid permease family protein |
| EF2712 | Hypothetical protein |
| EF2713 | Cell wall surface anchor family protein |
| EF2724 | Peptidase, M42 family |
| EF2735 | Exodeoxyribonuclease |
| EF2740 | O-methyltransferase family protein |
| EF2773 | Drug resistance transporter, putative, authentic frameshift |
| EF2794 | Membrane protein, putative |
| EF2795 | LysM domain lipoprotein |
| EF2860 | ErfK/YbiS/YcfS/YnhG family protein, putative |
| EF2870 | Conserved hypothetical protein, truncation |
| EF2879 | Acetyl-CoA carboxylase, biotin carboxyl carrier protein |
| EF2884 | Acyl carrier protein, putative |
| EF2904 | Hypothetical protein |
| EF2910 | Potassium uptake protein |
| EF2920 | ABC transporter, ATP-binding/permease protein |
| EF2927 | Hydrolase, haloacid dehalogenase-like family |
| EF2935 | Xanthine/uracil permeases family protein |
| EF2960 | Ribose transporter protein RbsD |
| EF2962 | Sugar-binding transcriptional regulator, LacI family |
| EF2963 | Esterase, putative |
| EF2968 | Cell wall surface anchor family protein |
| EF2970 | Conserved domain protein |
| EF2992 | Major facilitator family transporter |
| EF3003 | Lipoprotein, putative |
| EF3006 | Conserved hypothetical protein |
| EF3008 | Conserved hypothetical protein |
| EF3009 | Conserved hypothetical protein |
| EF3010 | Conserved hypothetical protein |
| EF3011 | Conserved hypothetical protein |
| EF3012 | Membrane protein, putative |
| EF3013 | Conserved domain protein |
| EF3019 | Conserved hypothetical protein |
| EF3022 | Sodium:dicarboxylate symporter family protein |
| EF3024 | Hypothetical protein |
| EF3029 | PTS system, IID component |
| EF3030 | PTS system, IIC component |
| EF3031 | PTS system, IIB component |
| EF3032 | Conserved hypothetical protein |
| EF3033 | PTS system, IIA component |
| EF3034 | Transcriptional regulator, GntR family |
| EF3046 | PTS system, IIA component |
| EF3047 | Conserved hypothetical protein |
| EF3074 | Hypothetical protein |
| EF3075 | Hypothetical protein |
| EF3076 | Cell wall surface anchor family protein |
| EF3084 | Iron compound ABC transporter, permease protein |
| EF3087 | Hypothetical protein |
| EF3095 | Hydrolase, haloacid dehalogenase-like family |
| EF3099 | Transporter accessory protein, putative |
| EF3101 | IS256, transposase |
| EF3102 | Hypothetical protein |
| EF3103 | Membrane protein, putative |
| EF3104 | ABC transporter, ATP-binding protein |
| EF3105 | Hypothetical protein |
| EF3127 | Guanylate kinase |
| EF3145 | Hypothetical protein |
| EF3146 | Hypothetical protein |
| EF3153 | Conserved hypothetical protein |
| EF3154 | Conserved hypothetical protein |
| EF3155 | Conserved hypothetical protein |
| EF3161 | Hypothetical protein |
| EF3199 | ABC transporter, permease protein |
| EF3204 | Cobalamin synthesis protein/P47K family protein |
| EF3207 | Dihydrouridine synthase family protein |
| EF3217 | Helicase, putative |
| EF3218 | Mutator MutT protein, putative |
| EF3220 | Hypothetical protein |
| EF3221 | Transcriptional regulator, Cro/CI family |
| EF3222 | Hypothetical protein |
| EF3223 | Hypothetical protein |
| EF3224 | Hypothetical protein |
| EF3225 | Conserved hypothetical protein |
| EF3226 | Rep protein |
| EF3227 | Conserved hypothetical protein |
| EF3230 | Conserved domain protein, authentic point mutation |
| EF3241 | Abortive phage resistance protein, putative |
| EF3242 | Abortive phage resistance protein, putative |
| EF3243 | Hypothetical protein |
| EF3245 | Cell-envelope associated acid phosphatase |
| EF3247 | Hypothetical protein |
| EF3248 | Hypothetical protein |
| EF3249 | Conserved hypothetical protein |
| EF3250 | Hypothetical protein |
| EF3251 | Hypothetical protein |
| EF3252 | Hypothetical protein |
| EF3253 | Cell wall surface anchor family protein |
| EF3261 | Transcriptional regulator, AbrB family |
| EF3262 | Transcriptional regulator, PemK family |
| EF3264 | Hypothetical protein |
| EF3272 | Zinc-binding transcriptional regulator, Cro/CI family |
| EF3273 | Hypothetical protein |
| EF3274 | Hypothetical protein |
| EF3294 | Membrane protein, putative |
| EF3302 | Hypothetical protein |
| EF3328 | Transcriptional regulator, GntR family |
